# Association of triglyceride glucose-body mass index with non-small cell lung cancer risk: A case-control study on Chinese adults

**DOI:** 10.3389/fnut.2022.1004179

**Published:** 2022-10-14

**Authors:** Feifei Wang, Ting He, Guoliang Wang, Tuo Han, Zhongqiang Yao

**Affiliations:** ^1^Department of Oncology, The 3201 Hospital Affiliated to Medical Department of Xi’an Jiaotong University, Hanzhong, Shaanxi, China; ^2^Department of Orthopedics, Second Affiliated Hospital, Air Force Medical University, Xi’an, Shaanxi, China; ^3^Department of Oncology Surgery, The 3201 Hospital Affiliated to Medical Department of Xi’an Jiaotong University, Hanzhong, Shaanxi, China

**Keywords:** triglyceride glucose-body mass index, non-small cell lung cancer, insulin resistance, triglyceride, glucose

## Abstract

**Background and objectives:**

Insulin resistance (IR) is closely related to non-small-cell lung cancer (NSCLC) risk. Recently, triglyceride glucose-body mass index (TyG-BMI) has been recognized as one of the simple indexes of insulin resistance (IR). However, there are limited data on the relationship between TyG-BMI and NSCLC. Here, we investigated the association of TyG-BMI with NSCLC risk in Chinese adults.

**Methods:**

This study consisted of 477 NSCLC cases and 954 healthy subjects. All participants were enrolled from 3201 Hospital affiliated to the Medical Department of Xi’an Jiaotong University. TyG-BMI was calculated based on the values of fasting blood glucose, triglyceride, and BMI. The association of TyG-BMI with NSCLC risk was estimated by logistic regression analysis.

**Results:**

The mean value of TyG-BMI was statistically increased in patients with NSCLC compared to the control group (201.11 ± 28.18 vs. 174 ± 23.78, *P* < 0.01). There was a significant positive association between TyG-BMI and NSCLC (*OR* = 1.014; 95% CI 1.007–1.021; *P* < 0.001) after controlling for confounding factors. Moreover, the prevalence of NSCLC was significantly elevated in participants in the high TyG-BMI tertiles than those in the intermediate and low TyG-BMI tertiles (60.46% vs. 12.61% vs. 26.83%, *P* < 0.01). Importantly, TyG-BMI achieved a significant diagnostic accuracy for NSCLC, with an AUC (area under the curve) of 0.769 and a cutoff value of 184.87.

**Conclusion:**

The findings suggest that TyG-BMI is a useful tool for assessing NSCLC risk. Thus, it is essential to follow up on high TyG-BMI, and lifestyle modification is needed to prevent NSCLC in people with high TyG-BMI.

## Introduction

Lung cancer has been alarmingly increasing and gaining more attention from many scholars, with non-small cell lung cancer (NSCLC) being the most common form (1). Smoking and environmental pollution have been recognized as two of the important risk factors of lung cancer, and interventions have been taken (2, 3). Unfortunately, the prevalence of lung cancer is still growing. Hence, a better understanding of other unknown risk factors of lung cancer is urgent.

Insulin resistance (IR), characterized by hyperinsulinemia, is involved in the physiopathologic mechanism of metabolic-related diseases such as obesity, metabolic syndrome, and non-alcoholic fatty liver disease (NAFLD) (4, 5). Moreover, some studies support the link between IR and cancer. Yin et al. showed that IR significantly increased thyroid cancer risk (6). Di Sebastiano et al. found a positive association of hyperinsulinemia with prostate cancer development, progression, and aggressiveness (7). Recently, the link between lung cancer and IR has also been investigated. However, conclusions from studies on the correlation between lung cancer and IR remained inconsistent (8–10). A case-cohort study conducted on men indicated that men with higher fasting insulin levels or IR had a significantly higher risk of lung cancer after multivariable adjustment (8). Another case-control study showed that a positive association was found between HOMA-IR (an insulin resistance index) and lung cancer. However, the positive association was not statistically significant when adjustment was made for leptin (9). Parekh et al. demonstrated that there was a stronger correlation between IR and increased risk of overall cancer mortality after excluding lung cancer deaths (10). The gold standard for measuring IR is hyperinsulinemic-euglycemic clamp testing, but because of its high cost and invasiveness, its clinical application is limited. HOMA-IR is widely conducted in the clinic to evaluate IR (11), but it is calculated based on serum insulin levels, which are usually determined for diabetes mellitus and are not suitable for the general population. Recently, triglyceride glucose-body mass index (TyG-BMI), which is calculated based on triglyceride (TG), fasting plasma glucose (FPG), and body mass index (BMI), has emerged as a powerful and simple tool to assess IR (12). TyG-BMI was even superior to the TyG index and its related parameters in predicting liver fibrosis (13). However, despite the known association between IR and cancer (6, 7), the association of NSCLC with TyG-BMI is not understood. We therefore aimed to investigate the association of TyG-BMI with NSCLC by performing a case-control study. Furthermore, we used receiver operating characteristic curves (ROC curves) to explore the possible utility of TyG-BMI as a predictive biomarker of NSCLC.

## Materials and methods

### Participants

A retrospective review of medical records of 510 patients with newly diagnosed NSCLC who were enrolled between October 2010 and September 2018 at 3201 Hospital affiliated to the Medical Department of Xi’an Jiaotong University was carried out. The eligible patients for this study had histological or cytological confirmation of NSCLC. Locally advanced diseases were based on clinical assessments. Information regarding risk factors and medical history was collected by face-to-face consultation. History of cancer, diabetes, coronary heart disease, cerebrovascular disease, and thyroid disease were the exclusion criteria. In addition, we also excluded participants with a history of some relevant medication use, such as immunosuppressant and fenofibrate triglyceride-lowering drugs, as well as women who were pregnant or breastfeeding. After applying the exclusion criteria, a total of 477 patients with NSCLC (the NSCLC group) were enrolled in our final analysis. For each case, two apparently healthy controls matched for sex and age who underwent physical examination in the same period were enrolled in the study. The exclusion criteria for the controls were self-reported medical history and were the same as those of the cases. Finally, 954 controls participated in this study. All the participants provided written informed consent. The study was approved by the Human Research Ethics Committee of 3201 Hospital affiliated to Medical Department of Xi’an Jiaotong University (approval number 2018005).

### Measurements and laboratory analysis

Clinical parameters and laboratory results were obtained from the medical records of all the subjects who were included in this study. Participants’ weights were measured with light indoor clothing, and their heights were measured with no shoes. BMI was then calculated as weight in kilograms divided by height in meters squared. Blood pressure was measured thrice with the participants in the sitting position after 5 min of rest.

Blood samples were collected from all participants following an overnight fasting for at least 12 hours and detected rapidly. Serum biochemical parameters, including FPG, TG, total cholesterol (TC), low-density lipoprotein cholesterol (LDL-C), high-density lipoprotein cholesterol (HDL-C), and uric acid, were determined with a biochemical autoanalyzer (Chemistry Analyzer Au5800; Olympus Medical Engineering Company, Japan). TyG-BMI was calculated according to the reference (14). White blood cell count (WBCC) and neutrophil count were determined using an automated blood cell counter (Beckman Coulter Ireland Inc. Mervue, Galway, Ireland).

### Statistical analysis

All statistics in the study were performed with the SPSS 18.0 statistical software. The demographic and clinical characteristics of the participants are presented as mean (standard deviation) or percentage (numbers). Differences between or among groups were compared by Student’s *t*-test, one-way ANOVA, or chi-square test. A binary logistic regression analysis was conducted to explore the independent risk factors for NSCLC. Moreover, we performed a receiver operating characteristic curve (ROC) analysis to evaluate the ability of TyG-BMI to predict NSCLC. The results were considered statistically significant at a two-tailed *P*-value of < 0.05.

## Results

### Baseline characteristics

The clinical characteristics of all the participants are described in Table 1. The patients with NSCLC were more likely to smoke and had higher BMI, WBCC, neutrophil count, FPG, TG, uric acid, and TyG-BMI, and lower TC, LDL-C, and HDL-C than the controls (all *P*< 0.05). However, no significant difference in age, sex, family history of lung cancer, and blood pressure was observed between the two groups. The patients with NSCLC had high TyG-BMI than the healthy controls in both men (201.99 ± 28.83 vs. 183.59 ± 24.5, *P*< 0.01) and women (199.99 ± 27.38 vs. 170.33 ± 22.46, *P*< 0.01).

**TABLE 1:** Demographic and clinical characteristics of all the participants.

	Control group (*n* = 954)	NSCLC group (*n* = 477)	*P*-value
Age (years)	59.02 ± 12.03	59.37 ± 10.65	0.32
Sex (male/female)	584/370	293/184	0.39
Smoking (*n*, %)	86 (9.01)	122 (25.58)	<0.01
Family history of lung cancer	14 (1.47)	8 (1.68)	0.41
SBP (mmHg)	129.11 ± 9.06	128.19 ± 7.29	0.26
DBP (mmHg)	84.05 ± 6.85	83.08 ± 7.02	0.22
BMI (kg/m^2^)	22.04 ± 2.67	23.79 ± 2.71	<0.01
WBCC(× 10^9/L)	5.29 ± 1.34	6.05 ± 1.90	<0.01
Neutrophil count (× 10^9/L)	3.12 ± 1.08	3.71 ± 1.64	<0.01
FPG (mmol/L)	4.95 ± 0.50	5.11 ± 0.97	0.01
TC (mmol/L)	4.93 ± 0.94	4.08 ± 0.84	<0.01
TG (mmol/L)	0.71 ± 0.21	1.30 ± 0.66	<0.01
LDL-C (mmol/L)	2.93 ± 0.69	2.39 ± 0.68	<0.01
HDL-C (mmol/L)	1.59 ± 0.33	1.06 ± 0.29	<0.01
Uric acid (μmol/L)	277.30 ± 70.67	331.92 ± 74.47	<0.01
TyG-BMI	174.00 ± 23.78	201.11 ± 28.18	<0.01

Values are presented as mean ± standard deviation. SBP, systolic blood pressure; DBP, diastolic blood pressure; BMI, body mass index; WBCC, white blood cell count; FPG, fasting plasma glucose; TC, total cholesterol; TG, triglyceride; LDL-C, low-density lipoprotein cholesterol; HDL-C, high-density lipoprotein cholesterol; TyG, triglyceride and glucose index.

### Association of triglyceride glucose-body mass index with non-small-cell lung cancer risk

We performed a binary logistic regression analysis of TyG-BMI and NSCLC risk. In the unadjusted model, TyG-BMI was positively correlated with NSCLC [odds ratio (OR) = 1.039; 95% confidence interval (CI) 1.034–1.044, *P* < 0.001].

**FIGURE 1:**
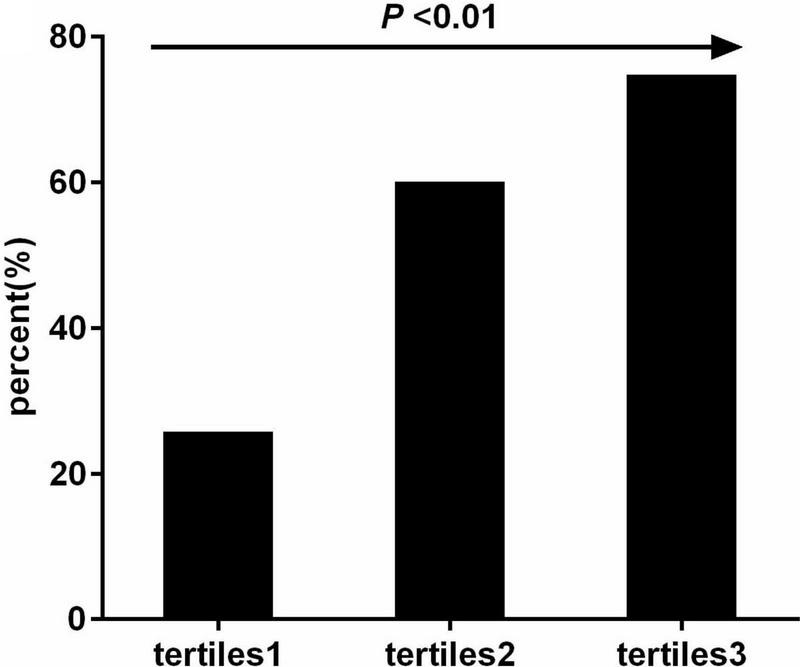
Prevalence of NSCLC according to the tertiles of TyG-BMI.

The OR (3.469; 95% CI 2.564–4.693, *P*< 0.001) for smoking as a risk factor for NSCLC was higher than that for TyG-BMI. After adjusting for potential covariates including lipid profile, TyG-BMI (OR = 1.014; 95% CI 1.007–1.021, *P*< 0.001) was still positively associated with NSCLC (Table 2). In addition, TyG-BMI was independently associated with NSCLC in both men (OR = 1.014; 95% CI 1.005–1.025, *P* = 0.004) and women (OR = 1.02; 95% CI 1.01–1.031, *P*< 0.001). Meanwhile, all the subjects were classified into tertiles according to TyG-BMI. The prevalence of NSCLC gradually increased as the TyG-BMI tertiles increased. The prevalence of NSCLC in participants in the high TyG-BMI tertiles was 60.46%, which showed a nearly fivefold increase compared with that in the low tertiles (Figure 1).

**TABLE 2:** Logistic regression analysis of TyG-BMI and NSCLC risk.

Model	*B*	*SE*	*Wald*	*OR*	*95%CI*	*P*
1	0.038	0.002	14.500	1.039	1.034–1.044	<0.001
2	0.014	0.004	15.081	1.014	1.007–1.021	<0.001

Model 1, unadjusted; model 2, adjustment made for age, sex, smoking, SBP, DBP, WBCC, neutrophil count, TC, LDL-C, HDL-C, and uric acid.

### The differences in triglyceride glucose-body mass index in different pathological or tumor-node-metastasis stages

According to the pathological stage, the patients with NSCLC were divided into the adenocarcinoma group (*n* = 397), the squamous cell carcinoma group (*n* = 66), and the other type group (*n* = 14). In addition, the patients with NSCLC were also divided into three groups: Tis (*n* = 72), TNM I (*n* = 237), and TNM II-IV (*n* = 168) according to tumor-node-metastasis (TNM) stage. There were no differences in TyG-BMI according to pathological (202.06 ± 27.31 vs. 196.72 ± 33.06 vs. 194.71 ± 26.92, *P* = 0.249) or TNM stage (197.74 ± 27.46 vs. 202.62 ± 26.87 vs. 200.4 ± 30.24, *P* = 0.404).

### Performance of triglyceride glucose-body mass index in predicting incident non-small-cell lung cancer

**FIGURE 2:**
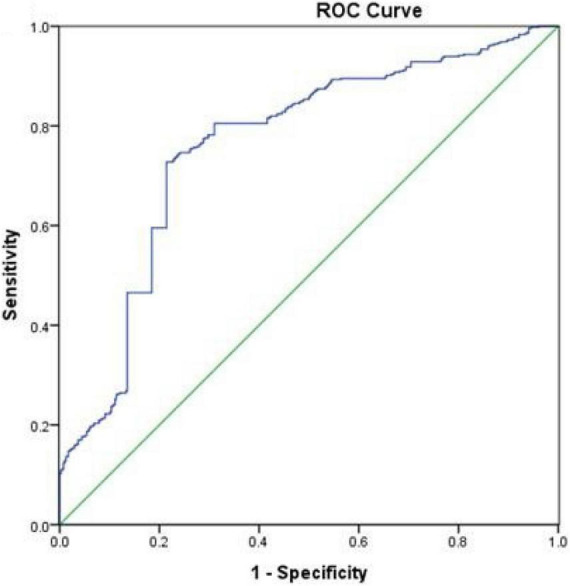
Receiver operative characteristic (ROC) curves of TyG-BMI for predicting the incidence of NSCLC.

Figure 2 presents the results of the ROC curve analyses of TyG-BMI for predicting incident NSCLC. TyG-BMI with a cut-off point of 184.87 showed a sensitivity of 72.5% and a specificity of 74.9% for predicting NSCLC, with 0.769 (95% CI 0.742–0.796, *P*< 0.001) AUROC. These results indicated that TyG-BMI might be regarded as an optimal marker for predicting the occurrence of NSCLC.

## Discussion

In the present study, we observed a strong and positive correlation between TyG-BMI and NSCLC risk even after controlling for confounding factors. However, there were no significant differences in the TyG-BMI levels of patients with different pathological or TNM stages. Moreover, TyG-BMI may be a simple and effective method for predicting incident NSCLC in Chinese adults.

Lung cancer is the major cause of both the incidence and mortality of all cancers in China (15). Unfortunately, the exact mechanisms responsible for lung cancer remain unclear. Recent epidemiological and clinical evidence points to an association of IR with the development and progression of cancer (16). A case–control study showed that HOMA-IR was significantly higher in patients with colorectal cancer than that in controls (17). A prospective study on postmenopausal women indicated that higher breast cancer incidence and all-cause mortality were observed in postmenopausal women with higher levels of IR (18). In addition, IR was also closely related to gastric cancer, prostate cancer, liver cancer, and endometrial cancer (19–21). Several published studies aiming to evaluate the association of lung cancer with IR drew inconsistent conclusions (8, 10). HOMA-IR calculated based on insulin levels is often used as an effective indicator for clinical evaluation of IR. However, plasma insulin levels are not often tested in the general population, especially in patients with cancer. Therefore, IR surrogate markers have been recently developed. The TyG index, which can be calculated from TG and FPG, has been proven to be a simple and effective tool to access IR (22). Several studies have demonstrated that the TyG index is better than HOMA-IR in predicting diseases such as arterial stiffness and non-alcoholic fatty liver (23, 24). In addition, a large prospective study from six European cohorts and a cross-sectional study from China indicated that the TyG index was correlated with increased risk of digestive system cancers and NSCLC (25, 26). TyG-BMI is another clinically useful marker for IR, as it combines TG, FPG, and BMI, and all the parameters are well confirmed for their roles in the development and progression of NSCLC (27, 28). To the best of our knowledge, no research has been performed to investigate the association of TyG-BMI with NSCLC risk. In the present study, we found that there was an independent correlation between TyG-BMI and NSCLC risk after adjusting for potential confounding factors. Besides, the prevalence of NSCLC gradually increased as the TyG-BMI tertiles increased. However, patients with NSCLC with different pathological or TNM stages had no differences in TyG-BMI. Of note, the current study first suggested that TyG-BMI was effective in distinguishing patients with NSCLC, in which TyG-BMI had a relatively larger AUC for predicting NSCLC. These findings highlighted the usefulness of this simple marker to identify individuals at high risk of developing an NSCLC event early in the Chinese population.

Although the mechanism involved in the link between NSCLC and TyG-BMI is poorly understood, IR may be involved in this relationship. On the one hand, hyperinsulinemia, which is a hallmark of IR, may contribute to the development of cancer because of the oncogenic potential of insulin by increasing the bioactivity of insulin-like growth factor I (IGF-I), enhancing growth factor-dependent cell proliferation, and/or directly affecting cell metabolism (29, 30). Furthermore, insulin-resistant patients have a high cancer risk, which may be due to excessive production of reactive oxygen species (ROS) (29). ROS can damage DNA, promote mutations, and cause cancer (31). On the other hand, TyG-BMI is based on adiposity, glucose, and TG. Obesity has been known to be a low-grade inflammatory state that can result in a protumorigenic environment (32). In addition, chronic hyperglycemia may also contribute to increased cancer risk (33). Of note, high levels of serum TG may promote the development of oxidative stress and ROS, thereby leading to proliferation and progression of cancer cells (34). Moreover, immunological modulations such as alteration in the proliferative capacity of lymphocytes and alteration in Th lymphocyte profile may participate in the mechanism (35).

Several limitations may exist in our study. First, this was a case-control study conducted to investigate the relationship between TyG-BMI and NSCLC. However, a longitudinal study is more convincing than a case control study. Second, a questionnaire was not delivered to collect information such as environmental pollution and lifestyle. Moreover, information about secondhand smoke and radiation to the chest has not been available in this study. By adjustment of multivariate factors, some important variables were missing. Third, since the study participants came from a single hospital, the generalizability of our study to the general population is uncertain.

## Conclusion

TyG-BMI is positively associated with NSCLC and a valuable marker for predicting the risk of NSCLC. This marker is easily calculated from routine laboratory data and anthropometric measurements, and we suggest the possibility of applying TyG-BMI to identify individuals at high risk of NSCLC in epidemiological surveys. Certainly, further prospective large-scale multicenter studies will be needed to confirm our results in the future.

## Data availability statement

The original contributions presented in the study are included in the article/supplementary material, further inquiries can be directed to the corresponding author/s.

## Ethics statement

The studies involving human participants were reviewed and approved by the Human Research Ethics Committee of 3201 Hospital. The patients/participants provided their written informed consent to participate in this study.

## Author contributions

FW and ZY participated in the study design, wrote and modified the manuscript, and prepared tables and figures. FW, THe, GW, and THa were involved in the conduct of the study and data collection. FW and THe made contributions to data analysis and results interpretation. All authors contributed to the article and approved the submitted version.
